# pyGenomeTracks: reproducible plots for multivariate genomic datasets 

**DOI:** 10.1093/bioinformatics/btaa692

**Published:** 2020-08-03

**Authors:** Lucille Lopez-Delisle, Leily Rabbani, Joachim Wolff, Vivek Bhardwaj, Rolf Backofen, Björn Grüning, Fidel Ramírez, Thomas Manke

**Affiliations:** UPDUB, ISREC Department, School of Life Sciences (SV), EPFL, 1015 Lausanne, Switzerland; Bioinformatics Group, Max Planck Institute of Immunobiology and Epigenetics, 79108 Freiburg, Germany; Bioinformatics Group, Department of Computer Science, University of Freiburg, 79110 Freiburg, Germany; Bioinformatics Group, Max Planck Institute of Immunobiology and Epigenetics, 79108 Freiburg, Germany; Bioinformatics Group, Department of Computer Science, University of Freiburg, 79110 Freiburg, Germany; Signalling Research Centres BIOSS and CIBSS, University of Freiburg, 79104 Freiburg, Germany; Bioinformatics Group, Department of Computer Science, University of Freiburg, 79110 Freiburg, Germany; Bioinformatics Group, Max Planck Institute of Immunobiology and Epigenetics, 79108 Freiburg, Germany; Bioinformatics Group, Max Planck Institute of Immunobiology and Epigenetics, 79108 Freiburg, Germany

## Abstract

**Motivation:**

Generating publication ready plots to display multiple genomic tracks can pose a serious challenge. Making desirable and accurate figures requires considerable effort. This is usually done by hand or using a vector graphic software.

**Results:**

pyGenomeTracks (PGT) is a modular plotting tool that easily combines multiple tracks. It enables a reproducible and standardized generation of highly customizable and publication ready images.

**Availability and implementation:**

PGT is available through a graphical interface on https://usegalaxy.eu and through the command line. It is provided on conda via the bioconda channel, on pip and it is openly developed on github: https://github.com/deeptools/pyGenomeTracks.

**Supplementary information:**

Supplementary data are available at *Bioinformatics* online.

## 1 Introduction

The analysis and visualization of multivariate genomic data faces several challenges. On one hand, there is a wide range of processing steps needed to analyze and to summarize large-scale data at a genome-wide level. Considerable effort has led to efficient tools as well as the adoption of scalable pipelines and frameworks, which provide a high degree of standardization and reproducibility (Bhardwaj *et al.*, 2019; Grüning *et al.*, 2018). On the other hand, advanced tools have been developed to support the visualization of genome-wide information and global patterns (Gehlenborg *et al.*, 2010). However, to turn genome-wide insights into testable interventions and validation experiments, researchers will usually return to locus-specific exploration. This is possible with a wide range of interactive genome browsers (Robinson *et al.*, 2011), and advanced browsers for three-dimensional data (Kerpedjiev *et al.*, 2018). Unfortunately, this exploration process is hard to standardize and yields heavily post-processed ‘snapshots’ to communicate the results. With pyGenomeTracks (PGT), we present a new and open software, which helps to standardize the generation of high-quality images in a programmatic approach. PGT supports the integrated visualization for a large variety of data sources, such as gene annotations, gene expression, chromatin signals and chromatin interactions.

## 2 Materials and methods

PGT provides an opportunity to map several genomic data tracks from a variety of resources onto one or a given list of genomic coordinates and generates an image per given coordinate including all of the input tracks. It offers support for a wide range of standard data formats in bioinformatics such as bigwig, bedgraph, epilogos, bed, gtf, narrow peaks, cool and HiCExplorer’s native h5 format.

The only preprocessing step to generate a multitracks plot is to prepare a configuration file which contains all necessary parameters to plot the desired tracks of multiple input files. PGT provides a simple script (*make_tracks_file*) to generate a configuration file from a collection of input files. A usage example of it is shown in Supplementary Section S1.

This configuration file defines best practice, but it can also be fully customized by the user. In a configuration file, each track is defined as a block of parameters starting with its name *[track name]* and continues with the parameters for that track such as the file location, its title, height, color and so on, as has been shown in the Supplementary Section S1.

For the plot generation, users need to define the precise genomic coordinates either by providing a single coordinate or by providing a bed file with multiple genomic regions. PGT supports several output formats such as *eps, pdf, pgf, png, ps, raw, rgba, svg* and *svgz*, which offers a broad degree of flexibility. The tool can easily generate the requested figure by running a single command line as has been presented below.

$ pyGenomeTracks ––tracks tracks.ini ––region \

chr2L:8050000-8300000 ––outFileName image.pdf

Moreover, for users who prefer a graphical interface, PGT is available as a tool on the European Galaxy server https://usegalaxy.eu, and can be installed on any local Galaxy instance (Afgan *et al.*, 2016) via ToolShed (see Supplementary Fig. S1).

**Fig. 1.**
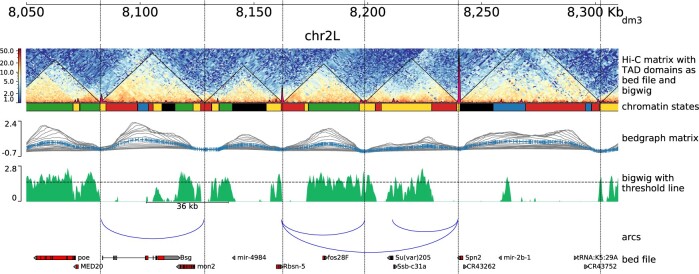
An example plot generated by PGT on *Drosophila melanogaster* (dm3) data, Kc167 cell line. The first track from the top shows the genomic locus (chromosome 2L 8.05–8.31 Mb). The second track illustrates a Hi-C matrix track (Li *et al.*, 2015) overlaid by its detected TADs, via HiCExplorer and a coverage profile of CP190 ChIP. The matrix was in HiCExplorer h5 format, TADs are given as a bed file which is a direct output of HiCExplorer’s hicFindTADs and the ChIP-Seq profile is provided as a bigwig file. The succeeding track shows the chromatin states, provided as a bed file, where the colors used are as defined in the ninth field of the bed file. The next track visualizes the TAD separation scores, the data are presented in a bedgraph matrix file format from HiCExplorer hicFindTADs. The green track shows a filled-out curve representation of the data from H3K36me3 histone mark, provided as a bigwig file along with an additional horizontal threshold line as well as a scale bar indicating the distance between two different peaks of interest. The blue arcs show artificially created links that could be contacts between different CP190 peaks. Finally, the last track is a gene track of dm3, available in bed format. The configuration file is available in Supplementary Section S3

To illustrate the functionality of PGT, Figure 1 provides an example of a multitrack visualization from an integrated multiomics screen (Ramírez *et al.*, 2018) generated with PGT version 3.5. Please refer to the Supplementary Data for additional examples and a detailed documentation is available on https://pygenometracks.readthedocs.io.

## 3 Conclusion

With PGT, it is possible to integrate multiple data sources from a wide variety of genomics assays and to generate publication ready plots. The presence of a configuration file (.ini file) provides flexibility to easily change or reorder the data tracks. To ensure maximal reproducibility, PGT also uses conda, which allows specific versions of all dependent tools to be flexibly chosen. This approach enables other researchers to readily reproduce the images and validate them swiftly. The supported output file formats, such as eps, svg or png, offer a high degree of freedom to generate plots in standardized formats which are required by a variety of major journals. PGT can be used as a command line or Galaxy-based tool. The latter is available on https://usegalaxy.eu with all configuration options, or it can be installed on any local Galaxy instance. It provides an easy way for users to run their analysis on Galaxy in a transparent and reproducible way. PGT presents a well-structured approach for generating genomics data plots and can also be used in automated workflow processing.

## Funding

This work was funded by the Deutsche Forschungsgemeinschaft (DFG, German Research Foundation) – Project-ID 192904750 – SFB 992. German Federal Ministry of Education and Research [031 A538A de. NBI-RBC awarded to R.B.; 031 L0101C de. NBI-epi awarded to B.G.]. R.B. was supported by the German Research Foundation (DFG) under Germany’s Excellence Strategy [CIBSS-EXC-2189-Project ID 390939984]. We acknowledge funding from the Ecole Polytechnique Fédérale (EPFL, Lausanne). LD was paid by the European Research Council (ERC) grant RegulHox (No 588029). 

*Conflict of Interest*: none declared.

## Supplementary Material

btaa692_Supplementary_Data
