# Supplementary material for: pyGenomeTracks: reproducible plots for multivariate genomic datasets
Source: Bioinformatics. 2020 Aug 3;37(3):422–3. doi: 10.1093/bioinformatics/btaa692 (PMC8058774; doi:10.1093/bioinformatics/btaa692)
Supplement: btaa692_Supplementary_Data [file btaa692_supplementary_data.pdf]

Supplementary material: pyGenomeTracks: Reproducible plots for multivariate genomic data sets

Lucille Lopez-Delisle[1], Leily Rabbani[2], Joachim Wolff[3], Vivek Bhardwaj[2], Rolf Backofen[3,4], Björn Grüning[3], Fidel Ramírez[2,*], and Thomas Manke[2]

[1]EPFL SV ISREC UPDUB, 1015 Lausanne, Switzerland
[2]Max Planck Institute of Immunobiology and Epigenetics, Stübeweg 51, 79108 Freiburg im Breisgau, Germany
[3] Bioinformatics Group, Department of Computer Science, University of Freiburg, Georges-Köhler-Allee 106, 79110 Freiburg, Germany
[4]Signalling Research Centres BIOSS and CIBSS, University of Freiburg, Schänzlestr. 18, 79104 Freiburg, Germany

*To whom correspondence should be addressed.

1 *make_tracks_file* or how to generate a configuration file

The script *make_tracks_file* can generate a configuration file from input file(s).

The full documentation is available on https://pygenometracks.readthedocs.io.

```
$ make_tracks_file --trackFiles file1.bed file2.bw -o tracks.ini
```

Will output with version 3.5

```
[x-axis]
#optional
#fontsize = 20
# default is bottom meaning below the axis line
# where = top

[spacer]
# height of space in cm (optional)
height = 0.5


[file1]
file = file1.bed

# title of track (plotted on the right side)
title = file1
# height of track in cm (ignored if the track is overlay on top the previous track)
height = 2
# if you want to plot the track upside-down:
# orientation = inverted
# if you want to plot the track on top of the previous track. Options are 'yes' or 'share-y'.
# For the 'share-y' option the y axis values is shared between this plot and the overlay plot.
# Otherwise, each plot use its own scale
#overlay_previous = yes

# If the bed file contains the exon
# structure (bed 12) then this is plotted. Otherwise
# a region **with direction** is plotted.
# If the bed file contains a column for color (column 9), then this color can be used by
# setting:
#color = bed_rgb
```

```
# if color is a valid colormap name (like RbBlGn), then the score (column 5) is mapped
# to the colormap.
# In this case, the the min_value and max_value for the score can be provided, otherwise
# the maximum score and minimum score found are used.
#color = RdYlBu
#min_value=0
#max_value=100
# If the color is simply a color name, then this color is used and the score is not considered.
color = darkblue
# whether printing the labels
labels = false
# optional:
# by default the labels are not printed if you have more than 60 features.
# to change it, just increase the value:
#max_labels = 60
# optional: font size can be given to override the default size
fontsize = 10
# optional: line_width
#line_width = 0.5
# the display parameter defines how the bed file is plotted.
# Default is 'stacked' where regions are plotted on different lines so
# we can see all regions and all labels.
# The other options are ['collapsed', 'interleaved', 'triangles']
# These options assume that the regions do not overlap.
# `collapsed`: The bed regions are plotted one after the other in one line.
# `interleaved`: The bed regions are plotted in two lines, first up, then down, then up etc.
# optional, default is black. To remove the border, simply set 'border_color' to none
# Not used in tssarrow style
#border_color = black
# style to plot the genes when the display is not triangles
#style = UCSC
#style = flybase
#style = tssarrow
# maximum number of gene rows to be plotted. This
# field is useful to limit large number of close genes
# to be printed over many rows. When several images want
# to be combined this must be set to get equal size
# otherwise, on each image the height of each gene changes
#gene_rows = 10
# by default the ymax is the number of
# rows occupied by the genes in the region plotted. However,
# by setting this option, the global maximum is used instead.
# This is useful to combine images that are all consistent and
# have the same number of rows.
#global_max_row = true
# If you want to plot all labels inside the plotting region:
#all_labels_inside = true
# If you want to display the name of the gene which goes over the plotted
# region in the right margin put:
#labels_in_margin = true
# if you use UCSC style, you can set the relative distance between 2 arrows on introns
# default is 2
#arrow_interval = 2
# if you use tssarrow style, you can choose the length of the arrow in bp
# (default is 4% of the plotted region)
#arrow_length = 5000
# if you use flybase or tssarrow style, you can choose the color of non-coding intervals:
#color_utr = grey
# as well as the proportion between their height and the one of coding
# (by default they are the same height):
#height_utr = 1
# By default, for oriented intervals in flybase style,
# or bed files with less than 12 columns, the arrowhead is added
```

```
# outside of the interval.
# If you want that the tip of the arrow correspond to
# the extremity of the interval use:
# arrowhead_included = true
# optional. If not given is guessed from the file ending.
file_type = bed

[file2]
file = file2.bw

# title of track (plotted on the right side)
title = file2
# height of track in cm (ignored if the track is overlay on top the previous track)
height = 2
# if you want to plot the track upside-down:
# orientation = inverted
# if you want to plot the track on top of the previous track. Options are 'yes' or 'share-y'.
# For the 'share-y' option the y axis values is shared between this plot and the overlay plot.
# Otherwise, each plot use its own scale
#overlay_previous = yes

color = #666666
# To use a different color for negative values
#negative_color = red
# To use transparency, you can use alpha
# default is 1
# alpha = 0.5
# the default for min_value and max_value is 'auto' which means that the scale will go
# roughly from the minimum value found in the region plotted to the maximum value found.
min_value = 0
#max_value = auto
# The number of bins takes the region to be plotted and divides it
# into the number of bins specified
# Then, at each bin the bigwig mean value is computed and plotted.
# A lower number of bins produces a coarser tracks
number_of_bins = 700
# to convert missing data (NaNs) into zeros. Otherwise, missing data is not plotted.
nans_to_zeros = true
# The possible summary methods are given by pyBigWig:
# mean/average/stdev/dev/max/min/cov/coverage/sum
# default is mean
summary_method = mean
# for type, the options are: line, points, fill. Default is fill
# to add the preferred line width or point size use:
# type = line:lw where lw (linewidth) is float
# similarly points:ms sets the point size (markersize (ms) to the given float
# type = line:0.5
# type = points:0.5
# set show_data_range to false to hide the text on the left showing the data range
show_data_range = true
# to compute operations on the fly on the file
# or between 2 bigwig files
# operation will be evaluated, it should contains file or
# file and second_file,
# we advice to use nans_to_zeros = true to avoid unexpected nan values
#operation = 0.89 * file
#operation = - file
#operation = file - second_file
#operation = log2((1 + file) / (1 + second_file))
#operation = max(file, second_file)
#second_file = path for the second file
# To log transform your data you can also use transform and log_pseudocount:
# For the transform values:
```

```
# 'log1p': transformed_values = log(1 + initial_values)
# 'log': transformed_values = log(log_pseudocount + initial_values)
# 'log2': transformed_values = log2(log_pseudocount + initial_values)
# 'log10': transformed_values = log10(log_pseudocount + initial_values)
# '-log': transformed_values = - log(log_pseudocount + initial_values)
# For example:
#tranform = log
#log_pseudocount = 2
# When a transformation is applied, by default the y axis
# gives the transformed values, if you prefer to see
# the original values:
#y_axis_values = original
# If you want to have a grid on the y-axis
#grid = true
file_type = bigwig
```

2 Galaxy wrapper

A history where you can see an example of pyGenomeTracks inputs and outputs is available at https://usegalaxy.eu/u/ldelisle/h/last-example-of-pgt.

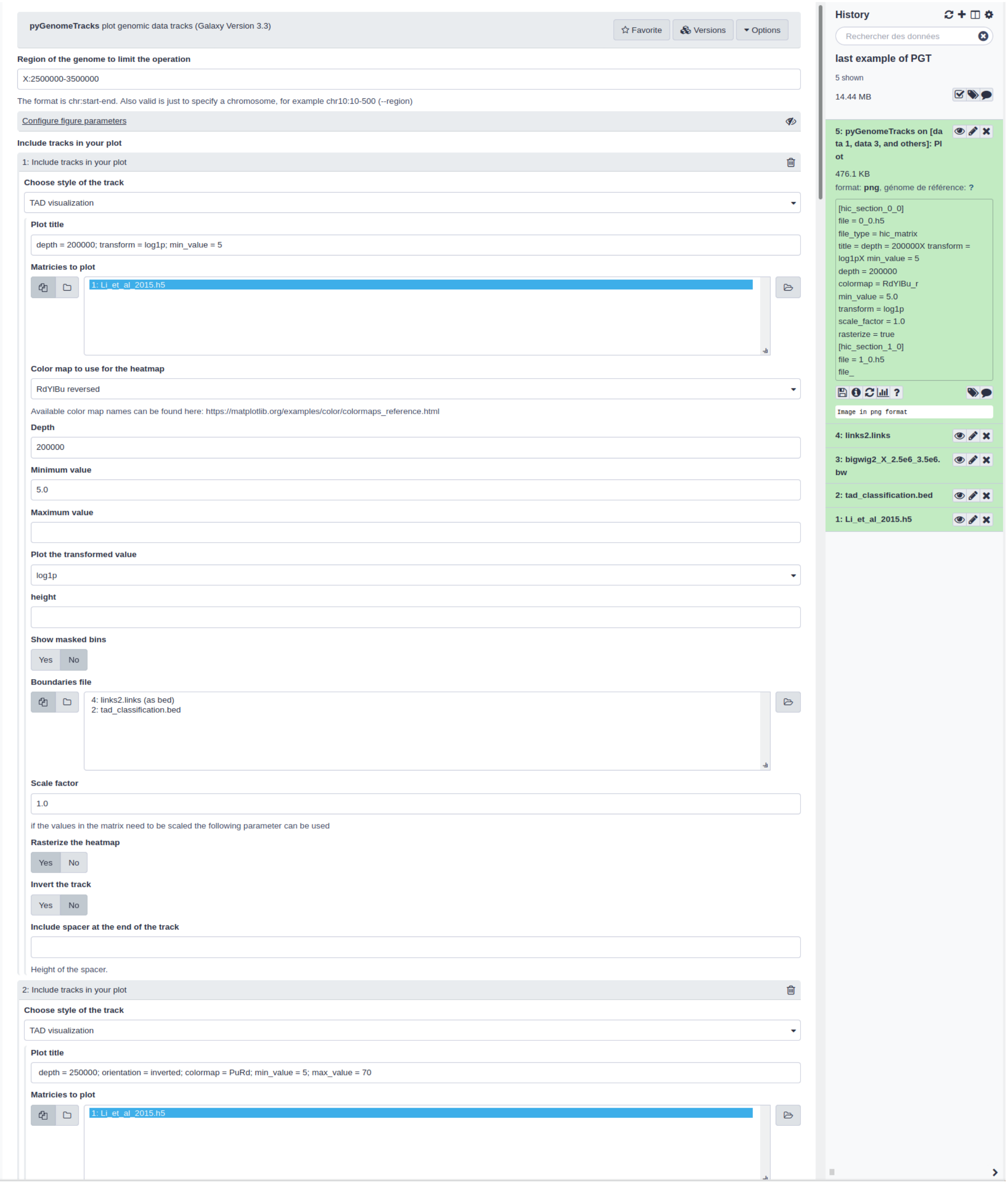

Figure 1: pyGenomeTracks wrapper for Galaxy.

3 Track file for the main figure

All used data is provided on zenodo: https://doi.org/10.5281/zenodo.3775381.

```
[x-axis]
fontsize = 20
title = dm3
```

```
[spacer]
height = 0.3

[HiC_Li_cubenas_et_al]
file = HiC_Cubenas.h5
height = 3
title = Hi-C matrix with TAD domains as bed file and bigwig
depth = 50000
transform = log1p
show_masked_bins = false
file_type = hic_matrix

[tad_classification]
file = tad__domains.bed
overlay_previous = share-y
color = none
height = 4
labels = false
fontsize = 10
file_type = domains

[CP190]
file = CP190.bw
overlay_previous = yes
show_data_range = false
height = 2
color = #FF007F
min_value = 0
number_of_bins = 700
nans_to_zeros = true
summary_method = mean
show_data_range = false
file_type = bigwig


[CP190_2]
file = CP190.bw
overlay_previous = yes
show_data_range = false
height = 2
color = #000000
min_value = 0
number_of_bins = 700
nans_to_zeros = true
summary_method = mean
type = line:0.75
show_data_range = false
file_type = bigwig


[chromatinStates_kc]
file = chromatinStates_kc.bed
title = chromatin states
height = 1
color = bed_rgb
display = collapsed
height = 0.5
labels = false
fontsize = 10
file_type = bed
show_data_range = false
```

```
[spacer]
height = 0.5

[tad_score]
file = tad__tad_score.bm
title = bedgraph matrix
color = none
height = 2
labels = false
fontsize = 10
type = lines
file_type = bedgraph_matrix

[spacer]
height = 0.5

[H3K36me3]
file = H3K36me3.bw
title = bigwig with threshold line
height = 2
color = #18B463
min_value = 0
number_of_bins = 700
nans_to_zeros = true
summary_method = mean
show_data_range = true
file_type = bigwig


[hlines]
file_type = hlines
y_values = 1.5
line_style = dashed
line_width = 1
overlay_previous = share-y
show_data_range = False

[scalebar]
file_type = scalebar
x_center = 8120730
size = 36000
where = bottom

[spacer]
height = 0.5

[vlines]
file = tad__domains.bed
type = vlines

[test arcs]
file = test.arcs
title = arcs
orientation = inverted
line_style = solid
height = 2

[genes]
file = dm3_genes_compact_no_cg.bed
height = 1
title = bed file
fontsize = 10
```

```
file_type = bed
gene_rows = 2
line_width = 0.5
color = red
```

4 Figure of the graphical abstract

The figure in the graphical abstract is more exhaustive than in the manuscript.

The first track from the top shows the genomic locus (chromosome 2L 8.05 Mb to 8.31 Mb). The second track illustrates a Hi-C matrix track (Li *et al.* (2015)) overlaid by its detected TADs, via HiCExplorer, and a coverage profile of CP190 ChIP. Although Hi-C tracks can be provided as cool Abdennur and Mirny (2019) or HiCExplorer's native h5 format (Ramírez *et al.* (2018)), here a matrix of h5 format has been used. TADs are given as a bed file which is a direct output of HiCExplorer's hicFindTADs, the ChIP-Seq profile is provided as a bigwig file (both Kent *et al.* (2010)). This track is followed by an inverted Hi-C matrix in h5 format (Cubeñas-Potts *et al.* (2017)). The interaction patterns in different conditions can be compared using this method. The succeeding track shows the chromatin states, provided as a bed file where the colors used are as defined in the 9th field of the bed file. The next track visualizes the TAD separation scores, the data is presented in a bedgraph matrix file format from HiCExplorer hicFindTADs. The green track shows a filled-out curve representation of the data from H3K36me3 histone mark, a mark which is correlated with the active chromatin state in *Drosophila melanogaster*, provided as a bigwig file. The following track shows another bigwig file as an orange line. The file contains the RNA polymerase II profile and the track has been plotted with an additional horizontal threshold line as well as a scale bar indicating the distance between two different peaks of interest. The blue arcs show artificially created links that could be contacts between different CP190 peaks. Finally the last track is a gene track of dm3. Although both gtf and bed formats (Karolchik *et al.* (2004)) are accepted by PGT, here a bed was used.

All used data is provided on zenodo: https://doi.org/10.5281/zenodo.3775381.

```
[x-axis]
fontsize = 20
title = dm3

[spacer]
height = 0.3

[HiC_Li_cubenas_et_al]
file = HiC_Cubenas.h5
height = 3
title = Hi-C matrix with TAD domains as bed file and bigwig
depth = 50000
transform = log1p
show_masked_bins = false
file_type = hic_matrix

[tad_classification]
file = tad__domains.bed
overlay_previous = yes
color = none
height = 4
labels = false
fontsize = 10
file_type = domains

[CP190]
file = CP190.bw
overlay_previous = yes
show_data_range = false
height = 2
color = #FF007F
min_value = 0
```

```
number_of_bins = 700
nans_to_zeros = true
summary_method = mean
show_data_range = false
file_type = bigwig


[CP190_2]
file = CP190.bw
overlay_previous = yes
show_data_range = false
height = 2
color = #000000
min_value = 0
number_of_bins = 700
nans_to_zeros = true
summary_method = mean
type = line:0.75
show_data_range = false
file_type = bigwig


[spacer]
height = 0.1

[HiC_cubenas_et_al]
file = HiC_Li_et_al.h5
title = inverted Hi-C matrix
depth = 50000
transform = log1p
show_masked_bins = false
file_type = hic_matrix
orientation = inverted
height = 3

[chromatinStates_kc]
file = chromatinStates_kc.bed
title = chromatin states
height = 1
color = bed_rgb
display = collapsed
height = 0.5
labels = false
fontsize = 10
file_type = bed
show_data_range = false


[spacer]
height = 0.5

[tad_score]
file = tad__tad_score.bm
title = bedgraph matrix
color = none
height = 2
labels = false
fontsize = 10
type = lines
file_type = bedgraph_matrix

[spacer]
height = 0.5
```

```
[H3K36me3]
file = H3K36me3.bw
title = bigwig
height = 2
color = #18B463
min_value = 0
number_of_bins = 700
nans_to_zeros = true
summary_method = mean
show_data_range = true
file_type = bigwig


[bigwig]
file = RNAPII.bw
title = bigwig with threshold and scalebar
type = line
color = orange
height = 3

[hlines]
file_type = hlines
y_values = 20
line_style = dashed
line_width = 1
overlay_previous = share-y

[scalebar]
file_type = scalebar
x_center = 8125730
size = 74770
where = bottom

[spacer]
height = 0.5

[vlines]
file = tad__domains.bed
type = vlines

[test arcs]
file = test.arcs
title = arcs
orientation = inverted
line_style = solid
height = 2

[genes]
file = dm3_genes_compact_no_cg.bed
height = 1
title = bed file
fontsize = 10
file_type = bed
gene_rows = 2
line_width = 0.5
color = red
```

5 Parameter supported for each track

Here is a table summarizing all the parameters supported for each track in version 3.5.

parameter	x_axis	epilogos	links	domains	bed	gtf	narrow_peak	bigwig	bedgraph	bedgraph_matrix	hlines	hic_matrix	scalebar
overlay_previous	X	X	X	X	X	X	X	X	X	X	X	X	X
where	X												X
fontsize	X				X	X							X
categories_file		X											
orientation		X	X	X	X	X	X	X	X	X	X	X	
links_type			X										
line_width			X	X	X	X	X				X		X
line_style			X								X		
color			X	X	X	X	X	X	X		X		X
alpha			X					X	X		X		X
max_value			X	X	X		X	X	X	X	X	X	
min_value			X	X	X			X	X	X	X	X	
ylim			X										
compact_arcs_level			X										
use_middle			X						X				
border_color				X	X	X							
prefered_name				X	X	X							
merge_transcripts				X	X	X							
labels					X	X							
style					X	X							
display					X	X							
max_labels					X	X							
global_max_row					X	X							
gene_rows					X	X							
arrow_interval					X	X							
arrowhead_included					X	X							
color_utr					X	X							
height_utr					X	X							
arrow_length					X	X							
all_labels_inside					X	X							
labels_in_margin					X	X							
show_data_range							X	X	X	X	X		
show_labels							X						
use_summit							X						
width_adjust							X						
type							X	X	X	X			
negative_color								X	X				
nans_to_zeros								X	X				
summary_method								X	X				
number_of_bins								X	X				
transform								X	X			X	
log_pseudocount								X	X				
y_axis_values								X	X				
second_file								X	X				
operation								X	X				
grid								X	X				
rasterize									X	X		X	
pos_score_in_bin										X			
plot_horizontal_lines										X			
colormap										X		X	
depth												X	
show_masked_bins												X	
scale_factor												X	
x_center													X
size													X

6 pyGenomeTracks examples

All used data is provided in our github repository:
https://github.com/deeptools/pyGenomeTracks/tree/master/examples and
https://github.com/deeptools/pyGenomeTracks/tree/master/pygenometracks/tests/test_data.

6.1 Basic examples

6.1.1 A bigwig track

```
[bigwig file test]
file = bigwig.bw
# height of the track in cm (optional value)
height = 4
title = bigwig
min_value = 0
max_value = 30
```

```
$ pyGenomeTracks --tracks bigwig_track.ini --region X:2,500,000-3,000,000 -o bigwig.png
```

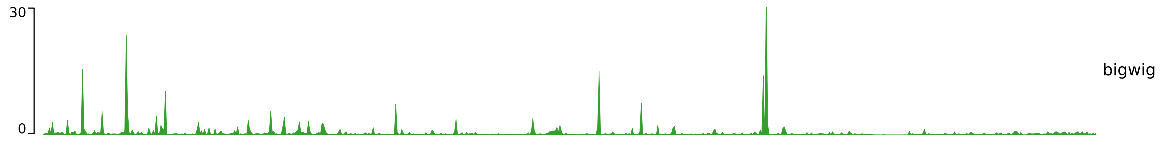

Figure 2: Bigwig track

6.1.2 Bigwig and genes

```
[bigwig file test]
file = bigwig.bw
# height of the track in cm (optional value)
height = 4
title = bigwig
min_value = 0
max_value = 30

[spacer]
# this simply adds an small space between the two tracks.

[genes]
file = genes.bed.gz
height = 7
title = genes
fontsize = 10
file_type = bed
gene_rows = 10

[x-axis]
fontsize=10
```

```
$ pyGenomeTracks --tracks bigwig_with_genes.ini --region X:2,800,000-3,100,000 -o bigwig_with_genes.eps
```

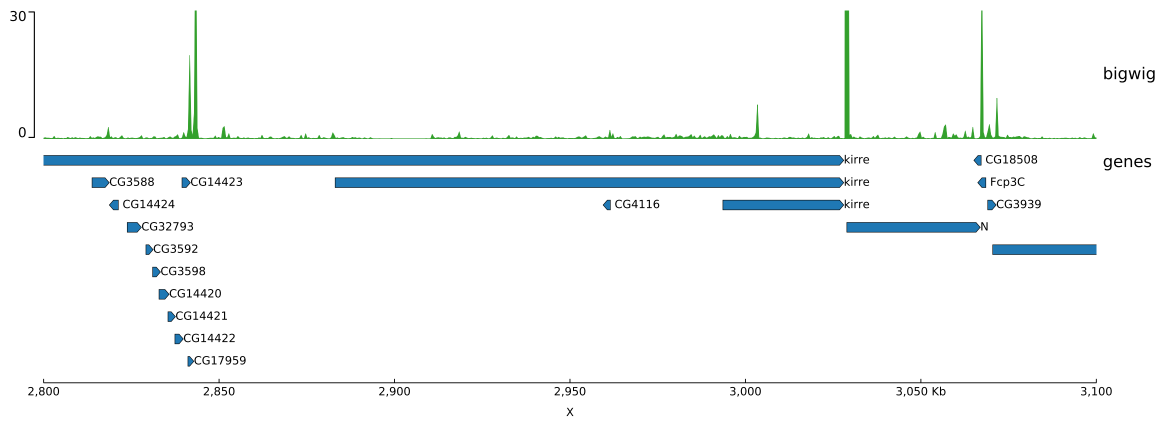

Figure 3: Bigwig and gene track

6.1.3 Bigwig, genes and vlines track

```
[bigwig file test]
file = bigwig.bw
# height of the track in cm (optional value)
height = 4
title = bigwig
min_value = 0
max_value = 30

[spacer]
# this simply adds an small space between the two tracks.

[genes]
file = genes.bed.gz
height = 7
title = genes
fontsize = 10
file_type = bed
gene_rows = 10

[x-axis]
fontsize=10

[vlines]
file = domains.bed
type = vlines
```

```
$ pyGenomeTracks --tracks bigwig_with_genes_and_vlines.ini --region X:2,800,000-3,100,000 -o
↪  bigwig_with_genes_and_vlines.eps
```

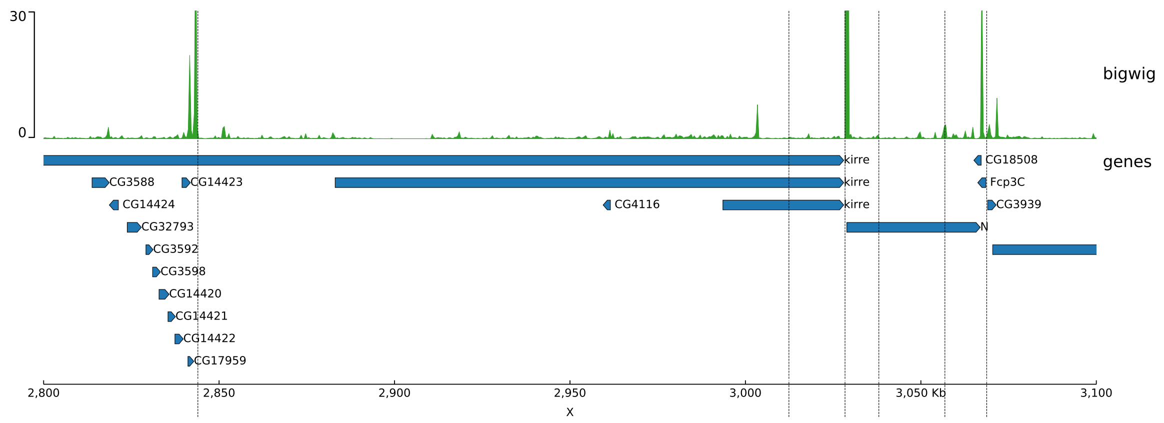

Figure 4: Bigwig, genes and vlines track

6.1.4 Bigwig overlay with transparency

```
[test bigwig]
file = bigwig2_X_2.5e6_3.5e6.bw
color = blue
height = 7
title = No alpha:
        (bigwig color=blue 2000 bins) overlaid with (bigwig color = (0.6, 0, 0) max over 300 bins) overlaid
↪  with (bigwig mean color = green 200 bins)
number_of_bins = 2000
min_value = 0
max_value = 30

[test bigwig max]
file = bigwig2_X_2.5e6_3.5e6.bw
color = (0.6, 0, 0)
summary_method = max
number_of_bins = 300
overlay_previous = share-y

[test bigwig mean]
file = bigwig2_X_2.5e6_3.5e6.bw
color = green
type = fill
number_of_bins = 200
overlay_previous = share-y

[spacer]

[test bigwig]
file = bigwig2_X_2.5e6_3.5e6.bw
color = blue
height = 7
title = alpha
        (bigwig color = blue 2000 bins) overlaid with (bigwig color = (0.6, 0, 0) alpha = 0.5 max over 300
↪  bins) overlaid with (bigwig mean color = green alpha = 0.5 200 bins)
number_of_bins = 2000
min_value = 0
max_value = 30

[test bigwig max]
file = bigwig2_X_2.5e6_3.5e6.bw
color = (0.6, 0, 0)
alpha = 0.5
summary_method = max
```

```
number_of_bins = 300
overlay_previous = share-y

[test bigwig mean]
file = bigwig2_X_2.5e6_3.5e6.bw
color = green
alpha = 0.5
type = fill
number_of_bins = 200
overlay_previous = share-y

[spacer]

[test bigwig]
file = bigwig2_X_2.5e6_3.5e6.bw
height = 7
title = alpha for lines/points:
        (bigwig color=(0.6, 0, 0) alpha = 0.5 max) overlaid with (bigwig mean color = green alpha = 0.5 line:2)
↪  overlaid with (bigwig min color = blue alpha = 0.5 points:2)
color = (0.6, 0, 0)
alpha = 0.5
summary_method = max
number_of_bins = 300
min_value = 0
max_value = 30

[test bigwig mean]
file = bigwig2_X_2.5e6_3.5e6.bw
color = green
type = line:2
alpha = 0.5
summary_method = mean
number_of_bins = 300
overlay_previous = share-y

[test bigwig min]
file = bigwig2_X_2.5e6_3.5e6.bw
color = blue
summary_method = min
number_of_bins = 1000
type = points:3
alpha = 0.5
overlay_previous = share-y

[x-axis]
```

```
$ pyGenomeTracks --tracks alpha.ini --region X:2700000-3100000 --trackLabelFraction 0.2 --dpi 130 -o
↪  master_alpha.png
```

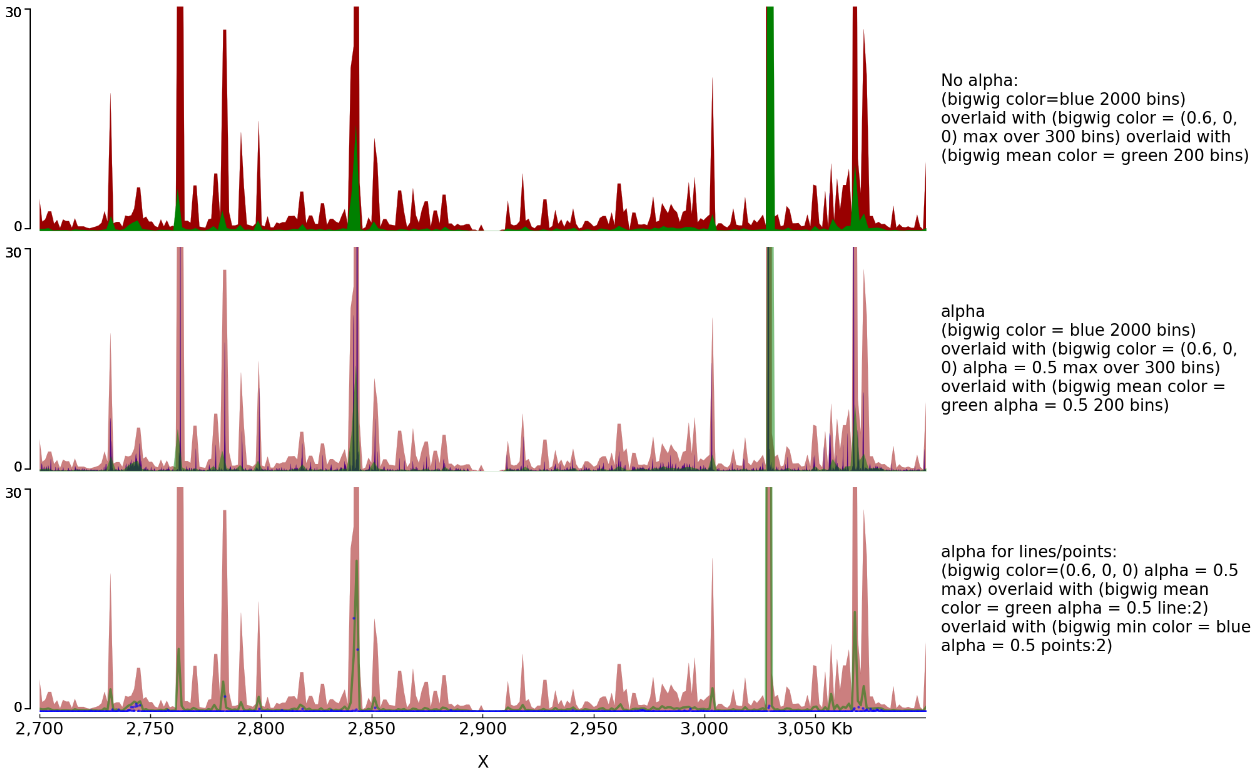

Figure 5: Bigwig overlay with transparency

6.2 Examples with bed and gtf

6.2.1 Bed and gtf format tracks

```
[x-axis]
where = top
title = where =top

[spacer]
height = 0.05

[genes 2]
file = dm3_genes.bed.gz
height = 7
title = genes (bed12) style = UCSC; fontsize = 10
style = UCSC
fontsize = 10

[genes 2bis]
file = dm3_genes.bed.gz
height = 7
title = genes (bed12) style = UCSC; arrow_interval=10; fontsize = 10
style = UCSC
arrow_interval = 10
fontsize = 10

[spacer]
height = 1

[test bed6]
file = dm3_genes.bed6.gz
height = 7
title = bed6 border_color = black; gene_rows=10; fontsize=7; color=Reds
```

```
        (when a color map is used for the color (e.g. coolwarm, Reds) the bed
        score column mapped to a color)
fontsize = 7
file_type = bed
color = Reds
border_color = black
gene_rows = 10

[spacer]
height = 1

[test bed4]
file = dm3_genes.bed4.gz
height = 10
title = bed4 fontsize = 10; line_width = 1.5; global_max_row = true
        (global_max_row sets the number of genes per row as the maximum found
        anywhere in the genome, hence the white space at the bottom)
fontsize = 10
file_type = bed
global_max_row = true
line_width = 1.5

[spacer]
height = 1

[test gtf]
file = dm3_subset_BDGP5.78.gtf.gz
height = 10
title = gtf from ensembl
fontsize = 12
file_type = bed

[spacer]
height = 1

[test bed]
file = dm3_subset_BDGP5.78_asbed_sorted.bed.gz
height = 10
title = gtf from ensembl in bed12
fontsize = 12
file_type = bed

[spacer]
height = 1

[test gtf collapsed]
file = dm3_subset_BDGP5.78.gtf.gz
height = 10
title = gtf from ensembl one entry per gene
merge_transcripts = true
prefered_name = gene_name
fontsize = 12
file_type = bed

[spacer]
height = 1

[x-axis]
fontsize = 30
title = fontsize = 30
```

```
$ pyGenomeTracks --tracks bed_and_gtf_tracks.ini --region X:3000000-3300000 --trackLabelFraction 0.2 --width 38
↪  --dpi 130 -o master_bed_and_gtf.eps
```

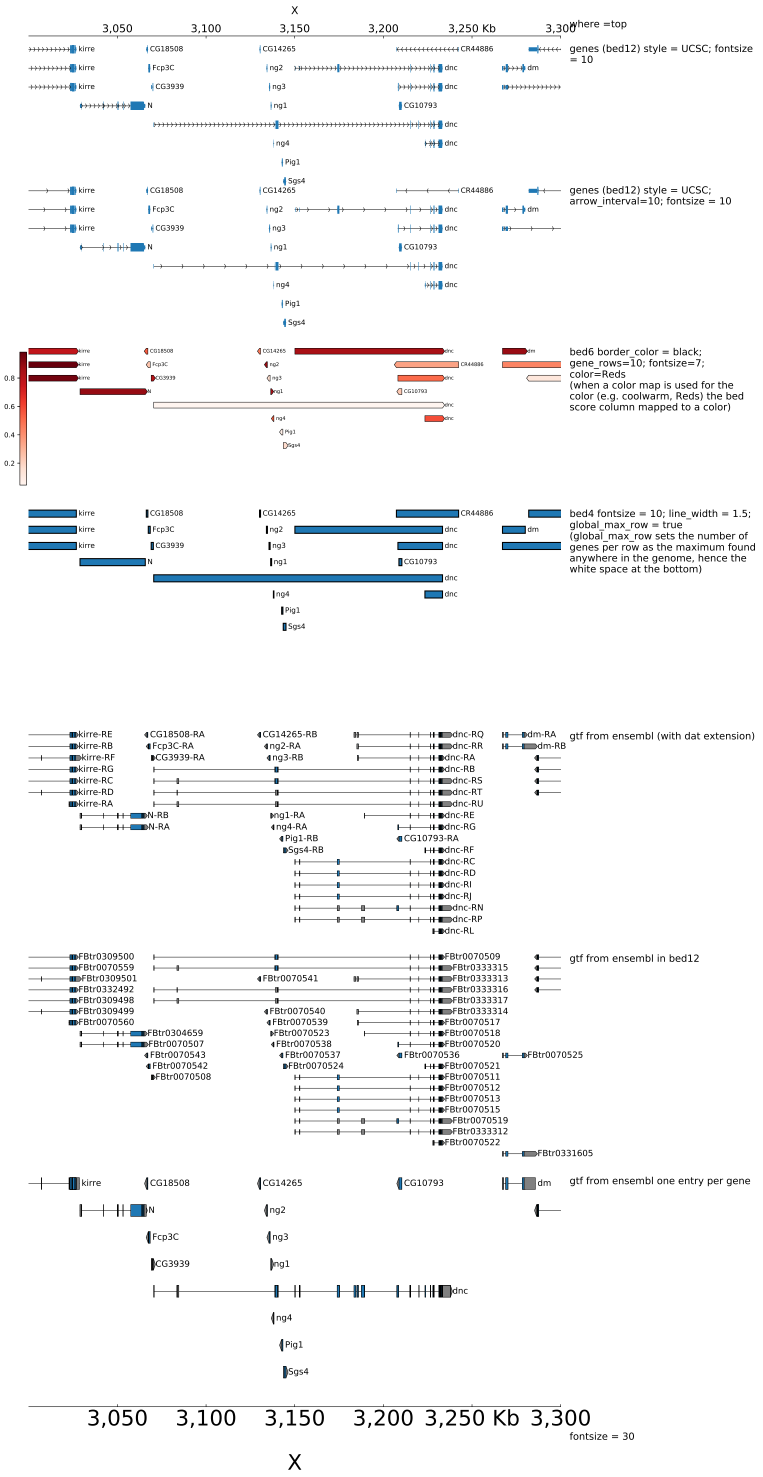

Figure 6: Bed and gtf format tracks

6.2.2 UTR settings

```
[x-axis]
where = top

[spacer]
height = 0.05

[genes 0]
file = dm3_genes.bed.gz
height = 7
title = genes (bed12) style = flybase; fontsize = 10
style = flybase
fontsize = 10

[spacer]
height = 1

[genes 1]
file = dm3_genes.bed.gz
height = 7
title = genes (bed12) style = flybase; fontsize = 10; color_utr = red
style = flybase
fontsize = 10
color_utr = red

[spacer]
height = 1

[genes 2]
file = dm3_genes.bed.gz
height = 7
title = genes (bed12) style = flybase; fontsize = 10; height_utr = 0.7
style = flybase
fontsize = 10
height_utr = 0.7
```

```
$ pyGenomeTracks --tracks bed_flybase_tracks.ini --region X:3000000-3300000 --trackLabelFraction 0.2 --width 38
↪  --dpi 130 -o master_bed_flybase.png
```

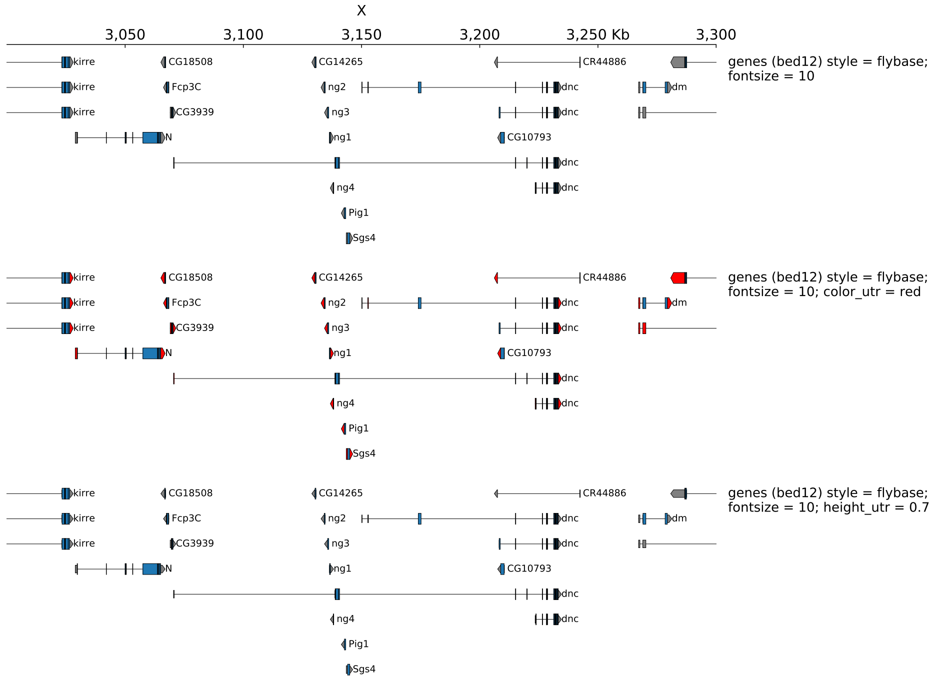

Figure 7: UTR

6.3 4C tracks

```
[x-axis]
where = top

[spacer]
height = 0.05

[test bedgraph]
file = GSM3182416_E12DHL_WT_Hoxd11vp.bedgraph.gz
color = blue
height = 5
title = bedgraph rasterize = true
rasterize = true
max_value = 10

[test bedgraph]
file = GSM3182416_E12DHL_WT_Hoxd11vp.bedgraph.gz
color = blue
height = 5
title = bedgraph
max_value = 10

[test bedgraph use middle]
file = GSM3182416_E12DHL_WT_Hoxd11vp.bedgraph.gz
color = blue
height = 5
title = bedgraph with use_middle = true
max_value = 10
use_middle = true

[genes]
file = HoxD_cluster_regulatory_regions_mm10.bed
height = 3
title = HoxD genes and regulatory regions
```

```
$ pyGenomeTracks --tracks bedgraph_useMid.ini --region chr2:74,000,000-74,800,000 --trackLabelFraction 0.2
↪  --width 38 --dpi 130  -o master_bedgraph_useMid_zoom.png
```

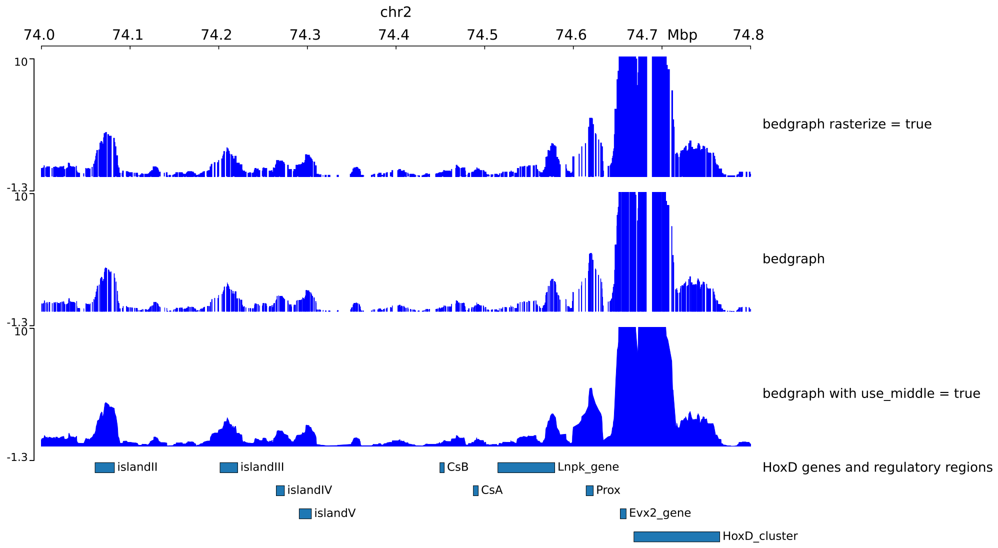

Figure 8: 4C track

6.4 Peaks

```
[narrow]
file = test2.narrowPeak
height = 4
max_value = 40
line_width = 0.1
title = max_value = 40;line_width = 0.1

[narrow 2]
file = test2.narrowPeak
height = 2
show_labels = false
show_data_range =  false
color = #00FF0080
use_summit = false
title = show_labels = false; show_data_range = false; use_summit = false; color = #00FF0080

[spacer]

[narrow 3]
file = test2.narrowPeak
height = 2
show_labels = false
color = #0000FF80
use_summit = false
width_adjust = 4
title = show_labels = false; use_summit = false; width_adjust = 4

[spacer]

[narrow 4]
file = test2.narrowPeak
height = 3
type = box
```

```
color = blue
line_width = 2
title = type = box; color = blue; line_width = 2

[spacer]

[narrow 5]
file = test2.narrowPeak
height = 3
type = box
color = blue
use_summit = false
title = type = box; color = blue; use_summit = false

[x-axis]
```

```
$ pyGenomeTracks --tracks narrow_peak2.ini --region X:2760000-2802000 --trackLabelFraction 0.2 --dpi 130 -o
↪  master_narrowPeak2.png
```

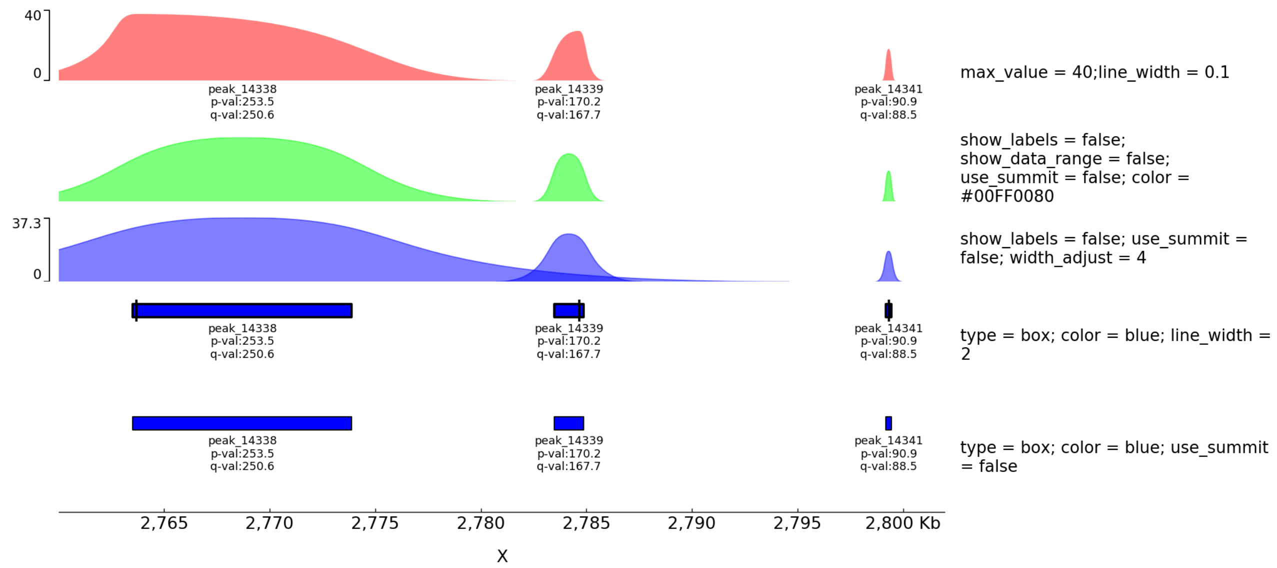

Figure 9: Peak track

6.5 Horizontal lines

```
[test hlines]
color = red
line_width = 2
line_style = dashed
y_values = 10, 200
min_value = 0
show_data_range = true
height = 5
title = hlines: color = red; line_width = 2; line_style = dashed; y_values = 10, 200
file_type = hlines

[spacer]

[test bigwig fill]
file = bigwig2_X_2.5e6_3.5e6.bw
```

```
color = gray
height = 2
type = fill
title = bigwig: gray fill overlayed with hlines at 10 and 200 blue dotted
max_value = 50

[test hlines ovelayed]
color = blue
line_style = dotted
y_values = 10, 200
overlay_previous = share-y
file_type = hlines

[spacer]

[x-axis]
```

```
$ pyGenomeTracks --tracks hlines.ini --region X:2700000-3100000 --trackLabelFraction 0.2 --dpi 130 -o
↪  master_hlines.png
```

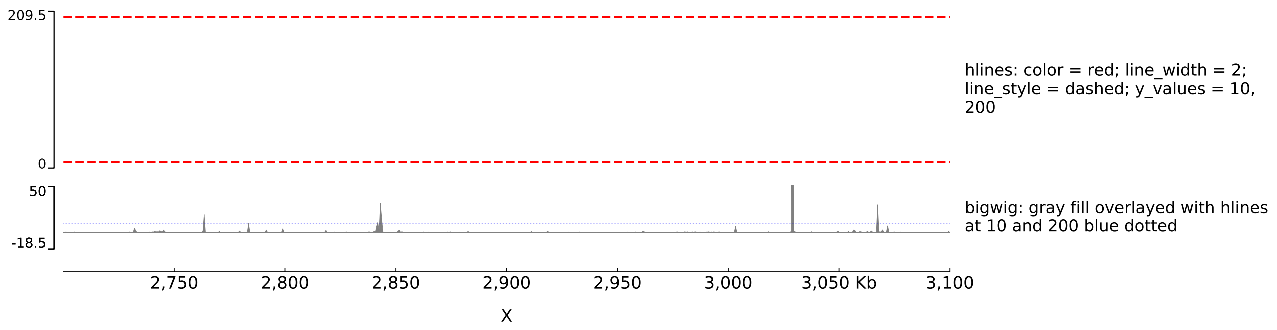

Figure 10: Horizontal lines track

6.6 Epilogos

```
[epilogos]
file = epilog.qcat.bgz
height = 5
title = height=5; categories_file=epilog_cats.json

[x-axis]
```

```
$ pyGenomeTracks  --tracks epilogos_track.ini --region X:3100000-3150000 -o epilogos_track.png
```

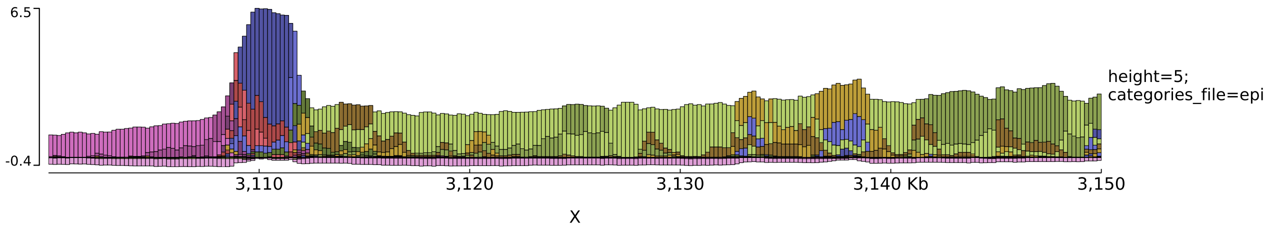

Figure 11: Epilogos track

6.6.1 Color setting

The color of the bars can be set by using a json file.

```
{
"categories":{
          "1":["Active TSS","#ff0000"],
          "2":["Flanking Active TSS","#ff4500"],
          "3":["Transcr at gene 5\" and 3\"","#32cd32"],
          "4":["Strong transcription","#008000"],
          "5":["Weak transcription","#006400"],
          "6":["Genic enhancers","#c2e105"],
          "7":["Enhancers","#ffff00"],
          "8":["ZNF genes & repeats","#66cdaa"],
          "9":["Heterochromatin","#8a91d0"],
          "10":["Bivalent/Poised TSS","#cd5c5c"],
          "11":["Flanking Bivalent TSS/Enh","#e9967a"],
          "12":["Bivalent Enhancer","#bdb76b"],
          "13":["Repressed PolyComb","#808080"],
          "14":["Weak Repressed PolyComb","#c0c0c0"],
          "15":["Quiescent/Low","#ffffff"]
        }
}
```

```
[epilogos]
file = epilog.qcat.bgz
height = 5
title = epilogos with custom colors
categories_file = epilog_cats.json

[epilogos inverted]
file = epilog.qcat.bgz
height = 5
title = epilogos inverted
orientation = inverted

[x-axis]
```

```
$ pyGenomeTracks  --tracks epilogos_track2.ini --region X:3100000-3150000 -o epilogos_track2.png
```

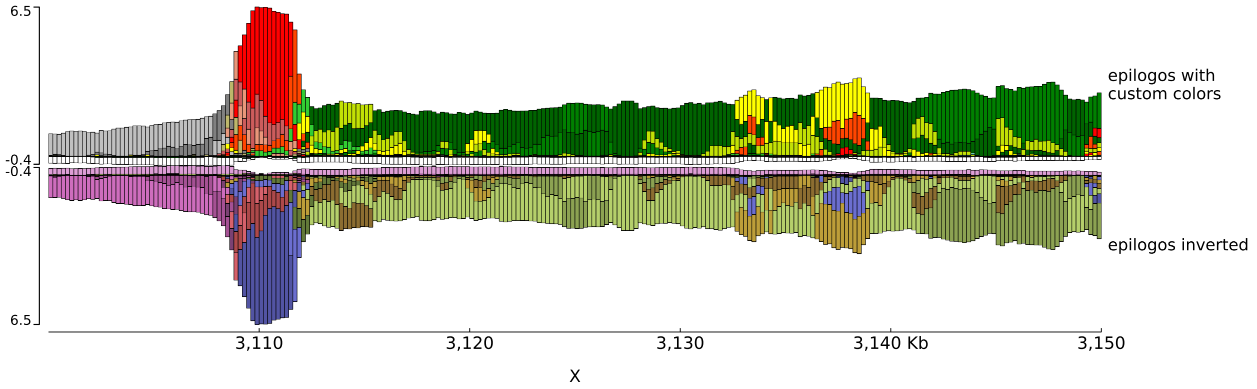

Figure 12: Epilogos track with color setting

6.7 Multiple combined tracks

```
[x-axis]
where = top
title = where=top

[spacer]
height = 0.05

[tads]
file = tad_classification.bed
title = TADs color = bed_rgb; border_color = black
file_type = domains
border_color = black
color = bed_rgb
height = 5

[tads 2]
file = tad_classification.bed
title = TADs orientation = inverted; color = #cccccc; border_color = red
file_type = domains
border_color = red
color = #cccccc
orientation = inverted
height = 3

[spacer]
height = 0.5

[tad state]
file = chromatinStates_kc.bed.gz
height = 1.2
title = bed display = interleaved; labels = false
display = interleaved
labels = false

[spacer]
height = 0.5

[tad state]
file = chromatinStates_kc.bed.gz
height = 0.5
title = bed display = collapsed; color = bed_rgb
labels = false
color = bed_rgb
display = collapsed

[spacer]
height = 0.5

[test bedgraph]
file = bedgraph_chrx_2e6_5e6.bg
color = blue
height = 1.5
title = bedgraph color = blue
max_value = 100

[test arcs]
file = test.arcs
title = links orientation = inverted
orientation = inverted
line_style = dashed
height = 2
```

```
[test bigwig]
file = bigwig2_X_2.5e6_3.5e6.bw
color = blue
height = 1.5
title = bigwig number_of_bins = 2000
number_of_bins = 2000

[spacer]

[test bigwig overlay]
file = bigwig2_X_2.5e6_3.5e6.bw
color = red
title = color:red; max_value = 50; number_of_bins = 100 (next track: overlay_previous = yes;
        max_value = 50; show_data_range = false; color = #0000FF80 (blue, with alpha 0.5))
min_value = 0
max_value = 50
height = 2
number_of_bins = 100

[test bigwig overlay]
file = bigwig_chrx_2e6_5e6.bw
color = #0000FF80
title =
min_value = 0
max_value = 50
show_data_range = false
overlay_previous = yes
number_of_bins = 100

[spacer]
height = 1

[tads 3]
file = tad_classification.bed
title = TADs color = #cccccc; border_color = red (next track:
        overlay_previous = share-y links_type = loops)
file_type = domains
border_color = red
color = #cccccc
height = 3

[test arcs overlay]
file = test.arcs
color = red
line_width = 10
links_type = loops
overlay_previous = share-y

[test arcs]
file = test.arcs
line_width = 3
color = RdYlGn
title = links line_width = 3 color RdYlGn
height = 3

[spacer]
height = 0.5
title = height = 0.5

[genes 2]
file = dm3_genes.bed.gz
height = 7
```

```
title = genes (bed12) style = flybase;fontsize = 10
style = flybase
fontsize = 10

[spacer]
height = 1

[test gene rows]
file = dm3_genes.bed.gz
height = 3
title = gene_rows = 3 (maximum 3 rows); style = UCSC
fontsize = 8
style = UCSC
gene_rows = 3

[spacer]
height = 1

[test bed6]
file = dm3_genes.bed6.gz
height = 7
title = bed6 border_color = black; gene_rows = 10; fontsize = 7; color = Reds
        (when a color map is used for the color (e.g. coolwarm, Reds) the bed
        score column mapped to a color)
fontsize = 7
file_type = bed
color = Reds
border_color = black
gene_rows = 10

[test bed6]
file = dm3_genes.bed6.gz
height = 10
title = bed6 fontsize = 10; line_width = 1.5; global_max_row = true
        (global_max_row sets the number of genes per row as the maximum found
        anywhere in the genome, hence the white space at the bottom)
fontsize = 10
file_type = bed
global_max_row = true
line_width = 1.5

[x-axis]
fontsize = 30
title = fontsize = 30

[vlines]
file = tad_classification.bed
type = vlines
```

```
$ pyGenomeTracks  --tracks browser_tracks.ini --region X:3000000-3500000 --trackLabelFraction 0.2 --width 38
↪  --dpi 130  -o master_plot.png
```

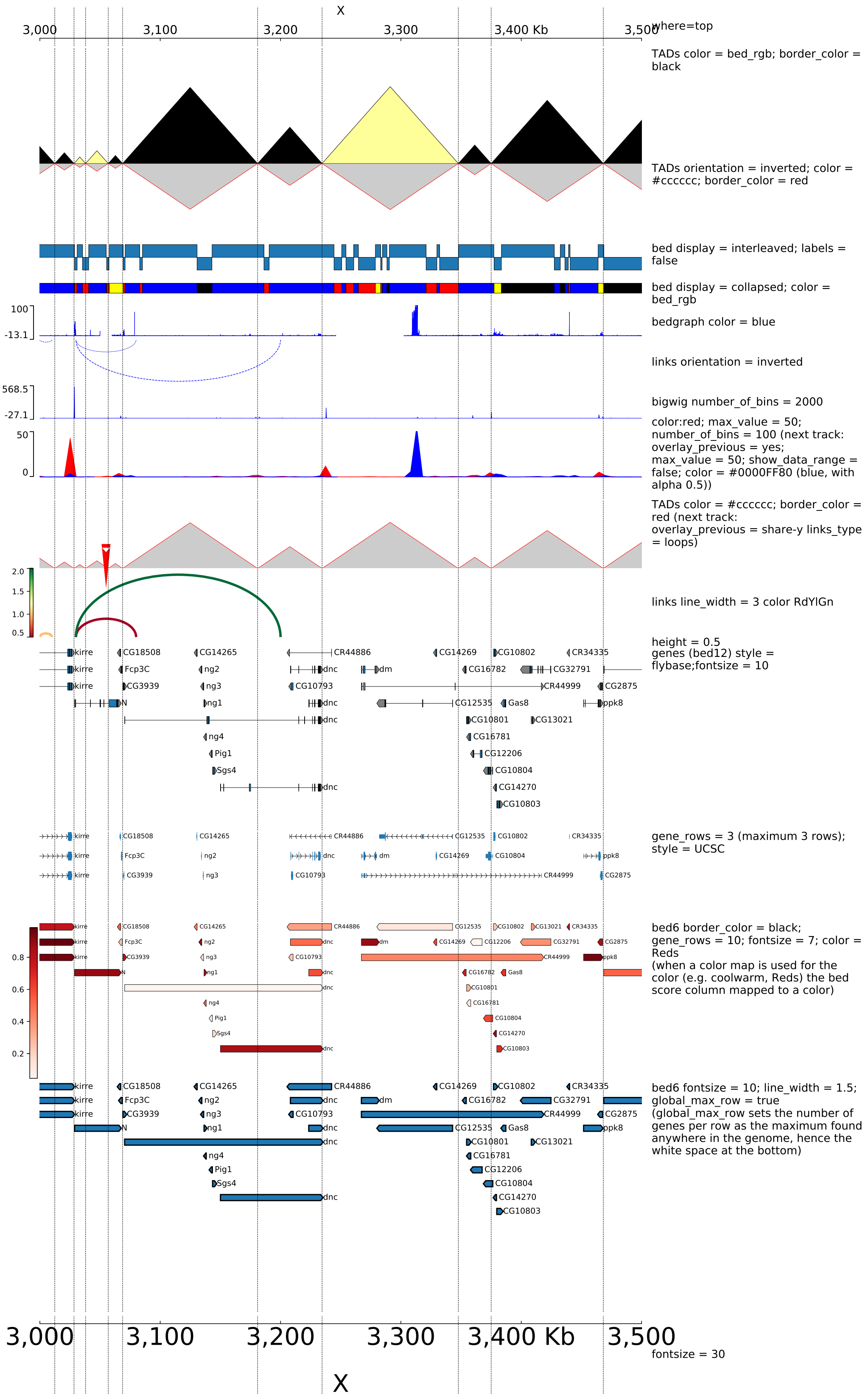

Figure 13: Multiple combined tracks

6.7.1 Multiple tracks with bigwigs

```
[test bigwig lines]
file = bigwig2_X_2.5e6_3.5e6.bw
color = gray
height = 2
type = line
title = orientation = inverted; show_data_range = false
orientation = inverted
show_data_range = false
max_value = 50

[test bigwig lines:0.2]
file = bigwig_chrx_2e6_5e6.bw
color = red
height = 2
type = line:0.2
title = type = line:0.2

[spacer]

[test bigwig points]
file = bigwig_chrx_2e6_5e6.bw
color = black
height = 2
min_value = 0
max_value = 100
type = points:0.5
title = type = point:0.5; min_value = 0; max_value = 100

[spacer]

[test bigwig nans to zeros]
file = bigwig_chrx_2e6_5e6.bw
color = red
height = 2
nans_to_zeros = true
title = nans_to_zeros = true

[spacer]

[test bigwig mean]
file = bigwig2_X_2.5e6_3.5e6.bw
color = gray
height = 5
title = gray:summary_method = mean; blue:summary_method = max;
        red:summary_method = min
type = line
summary_method = mean
max_value = 150
min_value = -5
show_data_range = false
number_of_bins = 300

[test bigwig max]
file = bigwig2_X_2.5e6_3.5e6.bw
#title = test
color = blue
type = line
summary_method = max
max_value = 150
min_value = -15
show_data_range = false
```

```
overlay_previous = share-y
number_of_bins = 300

[test bigwig min]
file = bigwig2_X_2.5e6_3.5e6.bw
color = red
type = line
summary_method = min
max_value = 150
min_value = -25
overlay_previous = share-y
number_of_bins = 300

[spacer]

[x-axis]
```

```
$ pyGenomeTracks  --tracks bigwig.ini --region X:2700000-3100000 --trackLabelFraction 0.2 --dpi 130 -o
↪  master_bigwig.png
```

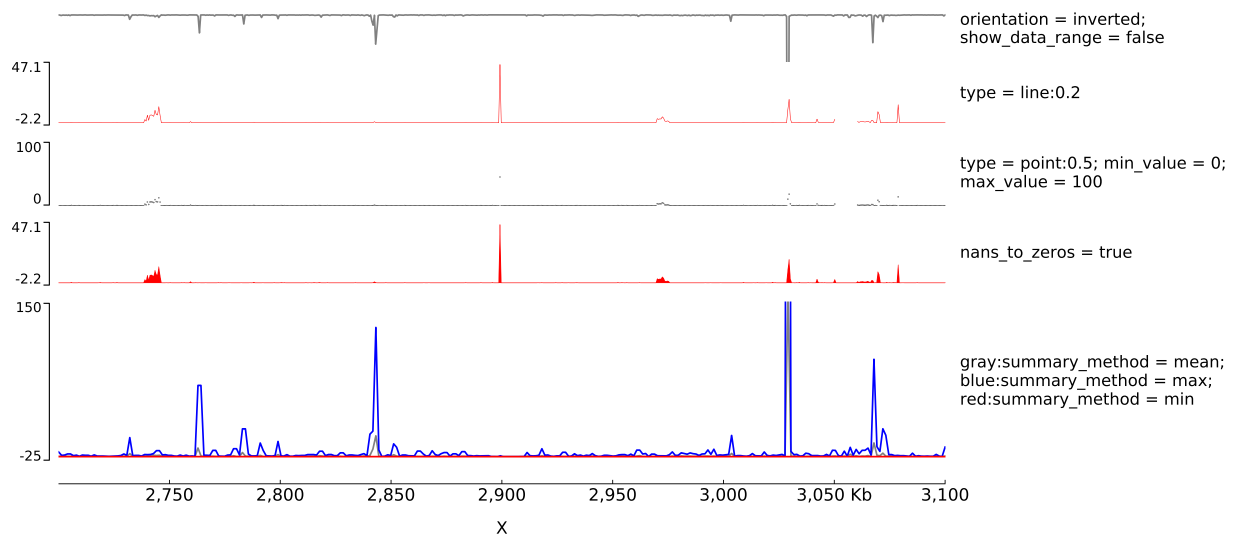

Figure 14: Multiple tracks with bigwigs

6.8 Hi-C tracks

```
[hic matrix]
file = Li_et_al_2015.h5
title = depth = 200000; transform = log1p; min_value = 5
depth = 200000
min_value = 5
transform = log1p
file_type = hic_matrix
show_masked_bins = false

[hic matrix]
file = Li_et_al_2015.h5
title = depth = 250000; orientation = inverted; colormap = PuRd; min_value = 5;
        max_value = 70
min_value = 5
```

```
max_value = 70
depth = 250000
colormap = PuRd
file_type = hic_matrix
show_masked_bins = false
orientation = inverted

[spacer]
height = 0.5

[hic matrix]
file = Li_et_al_2015.h5
title = depth = 300000; transform = log1p; colormap Blues (TADs:
        overlay_previous = share-y; line_width = 1.5)
colormap = Blues
min_value = 10
max_value = 150
depth = 300000
transform = log1p
file_type = hic_matrix

[tads]
file = tad_classification.bed
#title = TADs color = none; border_color = black
file_type = domains
border_color = black
color = none
height = 5
line_width = 1.5
overlay_previous = share-y
show_data_range = false

[spacer]
height = 0.5

[hic matrix]
file = Li_et_al_2015.h5
title = depth = 250000; transform = log1p; colormap = bone_r (links: overlay_previous = share-y;
        links_type = triangles; color = darkred; line_style = dashed, bigwig: color = red)
colormap = bone_r
min_value = 15
max_value = 200
depth = 250000
transform = log1p
file_type = hic_matrix
show_masked_bins = false

[test arcs]
file = links2.links
title =
links_type = triangles
line_style = dashed
overlay_previous = share-y
line_width = 0.8
color = darkred
show_data_range = false


[test bigwig]
file = bigwig2_X_2.5e6_3.5e6.bw
color = red
height = 4
title =
```

```
overlay_previous = yes
min_value = 0
max_value = 50
show_data_range = false

[spacer]
height = 0.5

[hic matrix]
file = Li_et_al_2015.h5
title = depth = 200000; show_masked_bins = true; colormap =
        ['blue', 'yellow', 'red']; max_value = 150
depth = 200000
colormap = ['blue', 'yellow', 'red']
max_value = 150
file_type = hic_matrix
show_masked_bins = true

[spacer]
height = 0.1

[x-axis]
```

```
$ pyGenomeTracks  --tracks browser_tracks_hic.ini --region X:2500000-3500000 --trackLabelFraction 0.23 --width
↪  38 --dpi 130 -o master_plot_hic.png
```

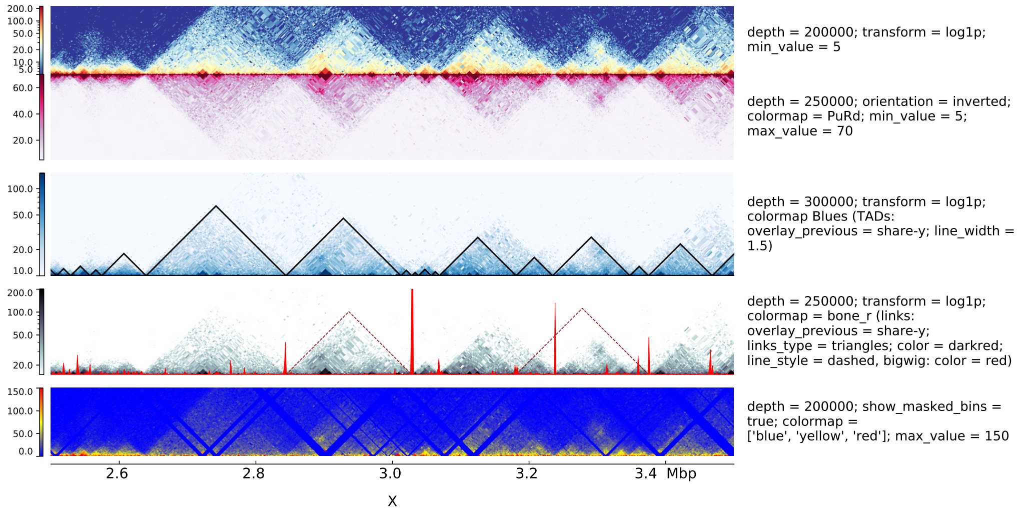

Figure 15: Hi-C tracks

References

Abdennur, N. and Mirny, L. A. (2019). Cooler: scalable storage for Hi-C data and other genomically labeled arrays. *Bioinformatics*, **36**(1), 311–316.

Cubeñas-Potts, C. *et al.* (2017). Different enhancer classes in drosophila bind distinct architectural proteins and mediate unique chromatin interactions and 3d architecture. *Nucleic acids research*, **45**(4), 1714–1730.

Karolchik, D. *et al.* (2004). The ucsc table browser data retrieval tool. *Nucleic acids research*, **32**(suppl_1), D493–D496.

Kent, W. J. *et al.* (2010). Bigwig and bigbed: enabling browsing of large distributed datasets. *Bioinformatics*, **26**(17), 2204–2207.

Li, L. *et al.* (2015). Widespread rearrangement of 3d chromatin organization underlies polycomb-mediated stress-induced silencing. *Molecular cell*, **58**(2), 216–231.

Ramírez, F. *et al.* (2018). High-resolution tads reveal dna sequences underlying genome organization in flies. *Nature communications*, **9**(1), 1–15.
